# Diagnosis of *Vespa affinis* venom allergy: use of immunochemical methods and a passive basophil activation test

**DOI:** 10.1186/s13223-019-0394-6

**Published:** 2019-12-04

**Authors:** Peshala Gunasekara, S. M. Handunnetti, Sunil Premawansa, Pradeep Kaluarachchi, Chandima Karunatilake, Indra P. Ratnayake, R. K. S. Dias, G. A. S. Premakumara, W. M. D. K. Dasanayake, Suranjith L. Seneviratne, Rajiva de Silva

**Affiliations:** 1 Institute of Biochemistry, Molecular Biology and Biotechnology, University of Colombo, Colombo 3, Sri Lanka; 2 Department of Zoology and Environment Sciences, Faculty of Science, University of Colombo, Colombo 3, Sri Lanka; 3 Healthcare Division, A. Baur & Co. (Pvt.) Ltd., No. 62, Jethawana Road, Colombo 14, Sri Lanka; 4 Department of Immunology, Medical Research Institute, Colombo 8, Sri Lanka; 5 District Hospital, Bandarawela, Sri Lanka; 6 Department of Zoology and Environmental Management, Faculty of Science, University of Kelaniya, Dalugama, Sri Lanka; 7 Department of Basic Sciences & Social Science, University of Colombo, Colombo, Sri Lanka; 8 Institute of Immunity and Transplantation, Royal Free Hospital and University College London, London, UK; 9 Department of Surgery, Faculty of Medicine, University of Colombo, Colombo 8, Sri Lanka

**Keywords:** CD63, IgE cross-reactivity, Insect venom allergy, Passive basophil activation test, *Vespa affinis*

## Abstract

**Background:**

Allergy to *Vespa affinis* venom is common in the Asia Pacific region. Venom preparations for diagnosis are not commercially available for this species.

**Methods:**

The prominent allergens in *V. affinis* venom were identifiedusing immunochemical methods. Use of ImmunoCAP of *Vespula vulgaris* crude venom/its components and a passive basophil activation test (BAT) in the diagnosis of patients who had anaphylaxis to *V. affinis* venom (n = 30) were also accessed. The IgE double-positivity rates (positive to both hornet and honeybee) in ImmunoCAP and the passive BAT were determined.

**Results:**

High IgE reactivity was seen with the five allergens in *V. affinis* venom; 96% (29/30) for 34 and 24 kDa, 93% (28/30) for 45 kDa and 90% (27/30) reactivity for the 100 and 80 kDa respectively. IgE cross-reactivity was low with ImmunoCAP using *V. vulgaris* venom (43%; 13/30) and Ves v1 (3%; 1/30), but relatively high with Ves v5 (73%; 22/30). All patients (100%) were positive to *V. affinis* venom in passive BAT. In ImmunoCAP, a high double-positivity rate (76%; 23/30) was detected while no double-positivity was detected in passive BAT.

**Conclusions:**

High IgE reactivity for five allergens of *V. affinis* points to the potential of using these allergens in component resolved diagnosis (CRD). The passive BAT has shown its importance as a promising diagnostic tool with high accuracy. It would be particularly useful in cases with doubtful double-positive results of other diagnostic tests.

## Background

*Vespa affinis* Linnaeus (*English: Lesser Banded Hornet, Sinhalese: Debara and Tamil: Kuḷavikaḷai*) is a hymenopteran insect of the family Vespidae, native to Sri Lanka and the Asia Pacific region [1, 2]. The distribution of *V. affinis* is confined to a small part of the world compared to *Vespula vulgaris*, the wasp prevalent in Western countries [3, 4]. IgE mediated hypersensitivity reactions to *V. affinis* stings is common in rural areas of Sri Lanka and is second only to *Apis dorsata* (Giant Asian Honeybee) among insect venom allergies in the country [5]. Unfortunately, in low income countries such as Sri Lanka, the incidence of stinging insect venom allergy is poorly documented and thus its adverse impact on the quality of health of the population may be underestimated [6–10]. In Sri Lanka, 6.7% of the patients (n = 30) had an anaphylactic shock after *V. affinis* sting [5]. A large case series from Vietnam has reported 16.3% of refractory hypotension and 6/43 (14%) of deaths after hornet sting allergic patients (n = 43) [8]. Fatalities due to anaphylaxis following *V. affinis*stings have been reported in Sri Lanka [11–13] and South East Asia [2, 14–17] Several case reports of *V. affinis* allergy and envenomation have also been reported from India [18–20], Nepal [21, 22] and Bangladesh [23, 24].

In vivo or in vitro diagnostics and venom specific immunotherapy are unavailable for *V. affinis* allergy, as pure or recombinant venom components are not commercially available. The basophil activation test (BAT) may be useful in the diagnosis of *V. affinis* venom allergy as basophils have surface markers which are upregulated following cross linking of surface IgE by cognate allergens [25]. The patient’s basophils presumably coated with venom specific IgE on its surface, are exposed to the culprit venom resulting in activation of the basophil. Activated basophils express activation markers on its surface (CD63 and/or CD203c) which can then be measured using flow cytometry. However, one of the major pitfalls of conventional BAT is that the analysis needs to be performed within 4 h of venipuncture [25]. This is not practical in countries such as Sri Lanka where most of the patients are from rural areas. Two studies showed that basophils from one individual could have their surface IgE replaced by IgE from a different donor [26, 27]. However, the passive BAT concept has not been proven for venom allergies. Therefore, our intention was to generate a passive BAT using donor basophils whose surface IgE have been removed and passively sensitized with IgE from venom allergic individuals. If an adequate activation could be demonstrated, the passive immune donor basophil activation test would be useful in the diagnosis of *V. affinis* venom allergy, and potentially, allergy to other venom species.

In addition, commercially available venom of closely related species (*Vespula vulgaris*) may be considered in the management of *V*. affinisvenom allergy, if high cross-reactivity could be determined. However, cross-reactivity due to cross-reactive carbohydrate determinants (CCD) would need to be excluded [28, 29]. True cross reactivity has been identified between two bee species; *Apis dorsata* (Giant Asian Honeybee) and *Apis mellifera* (Western honeybee) [30]. Characterization of the venom of *V. affinis* is still in its infancy when compared to *V. vulgaris* (wasp prevalent in Western countries) *Vespa crabro* L. (hornet in Western countries) or *Polybia paulista* I. (wasp prevalent in the Southern American region) [31–33]. Three immuno reactive proteins; Ves v1 (Phospholipase A_1_), Ves v2 (hyaluronidase) and Ves v5 (antigen 5) have been identified in *V. vulgaris* venom allergy and recombinant versions of Ves v1 and 5 are being incorporated in the diagnostic workflow [34]. Cross-reactivity between some venom components of *V. vulgaris, V. crabro* and *P. paulista* has been demonstrated [35, 36]. The identification of IgE reactive proteins in *V. affinis* venom and assessment of their cross-reactivity with the venom components of other species is essential for developing diagnostic workflows for *V. affinis* allergy which is prevalent in Asian countries.

The aims of our study were to (i) immunochemically characterize the prominent allergens in *V. affinis* venom (ii) assess the degree of IgE cross-reactivity with *V. vulgaris* crude venom and two recombinant components (rVes v1 and rVes v5), in the diagnosis of *V. affinis* venom allergy and (iii) assess the utility of the passive basophil activation test in the diagnosis of *V. affinis* venom allergy. Analyses were prospective and blinded.

## Methods

### Ethical clearance

Ethics approval for this study was obtained from the Ethics Review Committee, Medical Research Institute, Colombo, Sri Lanka (No: 46/2013). Permission to extract venom from *V. affinis* was obtained from the Department of Wildlife Conservation of Sri Lanka (WL/3/2/71/14).

### Patients

**Fig. 1:**
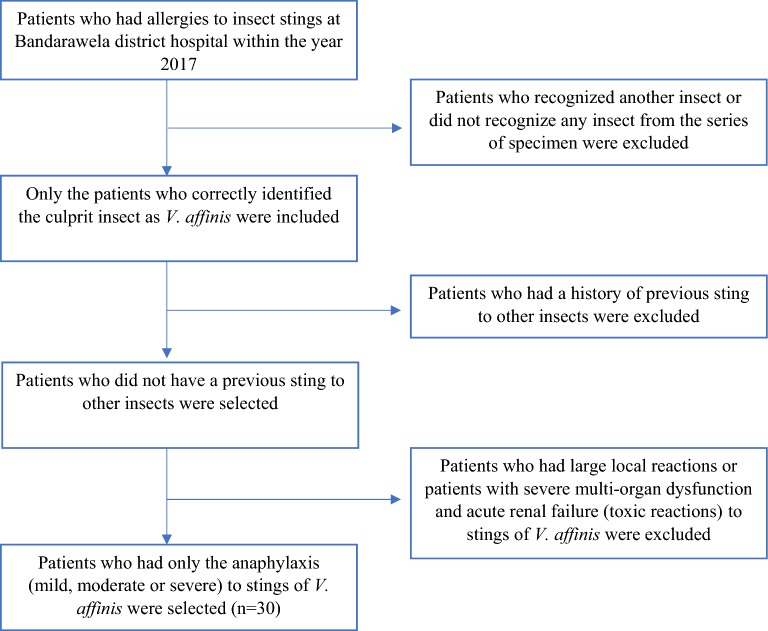
Flow chart of inclusion of the patients

Patients (n = 30) who developed anaphylaxis following a sting by *V. affinis* in 2017 at District Hospital, Bandarawela, Sri Lanka, were enrolled. Clinical details of the patients are shown in Table 1. The insect was identified by the specimens brought to the hospital by the patient and in the cases where the insect was not available, the patient was asked to identify the insect from a series of specimens of stinging hymenopterans. Only the patients who identified the culprit insect and did not have toxic reactions were selected through a questionnaire. The inclusion criteria were shown in Fig. 1. Patients with previous episodes of allergy/stings by other hymenoptera insects were excluded from further analysis. After the questionnaire was completed by the patient, 5 ml of blood was drawn within 1 h of hospitalization after obtaining informed written consent. After separation of the serum, the samples were kept at − 20 °C until further tests were carried out. Sera (from 5 ml of blood) of the patients (n = 30) who had anaphylaxis after *A. dorsata* (Giant Asian Honeybee) were also collected (using the same procedure) to compare the cross-reactivity by immunoblots.

**Table 1:** Serological data and Phadia ImmunoCAP test results of the patients selected

No	Age	Gender	Culprit insect (as identified by patient)	Severity	Phadia test (kU_A_/l; cut off > 0.1)			
					*Vespula vulgaris*	Ves V 1	Ves v 5	*Apis mellifera*
1	73	M	*V. affinis*	Moderate	1.08	Neg	26.3	3.55
2	51	M	*V. affinis*	Moderate	Neg	Neg	1.88	Neg
3	38	M	*V. affinis*	Mild	0.67	Neg	21.7	7.38
4	53	F	*V. affinis*	Moderate	0.68	Neg	Neg	4.99
5	57	M	*V. affinis*	Moderate	Neg	Neg	1.2	6.37
6	32	M	*V. affinis*	Moderate	1.18	Neg	27.4	17.3
7	61	M	*V. affinis*	Moderate	0.35	Neg	Neg	16.7
8	24	M	*V. affinis*	Moderate	Neg	Neg	5.39	Neg
9	34	M	*V. affinis*	Moderate	4.39	Neg	0.37	10
10	60	M	*V. affinis*	Moderate	Neg	Neg	36.9	1.62
11	61	M	*V. affinis*	Moderate	2.09	Neg	Neg	5.03
12	40	F	*V. affinis*	Moderate	Neg	Neg	Neg	0.45
13	54	F	*V. affinis*	Moderate	Neg	Neg	0.6	Neg
14	72	M	*V. affinis*	Moderate	6.7	Neg	4.37	27.2
15	52	M	*V. affinis*	Moderate	7.33	Neg	Neg	44.6
16	16	M	*V. affinis*	Moderate	Neg	Neg	2.45	3.68
17	42	M	*V. affinis*	Moderate	5.35	1.23	2.92	3.69
18	62	M	*V. affinis*	Moderate	Neg	Neg	13.4	1.87
19	75	F	*V. affinis*	Moderate	1.06	Neg	Neg	24
20	28	F	*V. affinis*	Moderate	Neg	Neg	12.6	5.38
21	72	F	*V. affinis*	Moderate	Neg	Neg	3.4	4.89
22	46	M	*V. affinis*	Moderate	Neg	Neg	0.52	Neg
23	69	M	*V. affinis*	Moderate	Neg	Neg	0.62	Neg
24	48	M	*V. affinis*	Moderate	Neg	Neg	2.01	Neg
25	54	F	*V. affinis*	Moderate	0.52	Neg	Neg	Neg
26	65	F	*V. affinis*	Moderate	Neg	Neg	47.1	4.21
27	62	M	*V. affinis*	Moderate	2.11	Neg	Neg	11.6
28	53	F	*V. affinis*	Moderate	Neg	Neg	1.44	9.21
29	61	M	*V. affinis*	Moderate	Neg	Neg	1.63	4.64
30	57	M	*V. affinis*	Moderate	Neg	Neg	1.58	1.36

Neg ≤ 0.1 kU_A_/l

### Insect identification and venom extraction

Insects were identified using their morphological characteristics as described previously [37]. Briefly, the presence of orange or red metasomal segments I and II followed by posterior black segments and ventrally well-defined, nearly contiguous metapleural punctures. The venom of *V. affinis* was extracted by electrical stimulation as described previously [30]. Briefly, *V. affinis* were stimulated using a voltage which could eject venom from the stinger on to a glass plate without killing the hornets. Dried venom on the glass plate was scraped out and dissolved in 0.13 M PBS. The protein content of the venom collected was determined using the Bradford method using bovine serum albumin (BSA) as the standard [38].

### Sodium dodecyl sulphate polyacrylamide gel electrophoresis (SDS-PAGE) and immunoblotting

SDS-PAGE was performed according to the method described previously [30]. Briefly, 10 µl of venom together with 5 µl of 3× Lammelli sample buffer was incubated at 95 °C for 5 min. It was added to each well of a 13.5% gel and electrophoresed at 70 V at 4 °C for 3 h. The gel was stained with Coomassie violet for 1 h and de-stained for about 5 h until the bands were cleared.

Immunoblotting was carried out to determine the specific IgE to venom components [30]. Briefly, the venom proteins were first separated by SDS-PAGE and were subsequently transferred to a nitrocellulose membrane using a mini protein tetra system (Bio-Rad). After blocking the membrane with PBST containing 5% nonfat milk at 4 °C for 1 h, it was reacted with 1:40 dilution of patient’s serum in antibody diluting buffer at 4 °C for overnight. After 3 subsequent washing steps with phosphate buffered saline (PBS) containing 0.05% Tween 20 (PBST), the membrane was then reacted at 4 °C for 2 h with a 1:1000 dilution of peroxidase-labelled goat anti-human IgE antibody (Sigma-Aldrich). The membrane was visualized using 4-cloro-naphthol substrate.

### Determination of hyaluronidase in HPLC fractions of *V. affinis* venom

The venom of *V. affinis* was analyzed using High Performance Liquid Chromatography (HPLC) using a similar solvent system and conditions described previously [30]. The fractions corresponding to each peak of the HPLC chromatogram were further tested for hyaluronidase activity to determine the hyaluronidase fraction. The purity of the fraction which responded to the enzyme activity assay was tested using SDS-PAGE and IgE reactivity was tested using immunoblots. To detect the IgE cross-reactivity to hyaluronidase, immunoblots in both directions were carried out with sera from either *V. affinis* (n = 30) or *A. dorsata* (n = 30) venom allergic patients. Four healthy control sera were used as controls.

### Hyaluronidase activity of the venom of *V. affinis* and *A. dorsata*

Hyaluronidase enzyme activity was determined using a turbidimetric method as described previously [30]. Briefly, the venom of *V. affinis* or *A. dorsata* with the different venom concentrations; 1, 2.5, 5, 7.5, 10, 15, 20, and 25 μg/ml were diluted in 50 μl of sodium acetate buffer (0.2 M sodium acetate, 0.15 M NaCl, pH 6.0). It was then incubated with 50 μl of the hyaluronic acid substrate at 37 °C for 15 min. After the incubation, 900 µl of bovine serum albumin (BSA) in acetate buffer (pH 3) was added to the samples. The resulting turbidity was read at 540 nm in a microplate reader after 5 min of incubation at room temperature. This was repeated using hyaluronidase fractions eluted from HPLC.

### Immunoblot inhibition assay

Immunoblot inhibition assay was carried out by a method described previously [30]. Four concentrations (15.62, 31.25, 62.5 and 125 µg) of *A. dorsata* venom were pre-incubated with 2 ml of antibody diluting buffer containing the pooled serum from 16 patients and separately serum from eight individual patients for 2 h with gentle agitation. The sera were then incubated with strips containing *V. affinis* venom according to the methods described in a previous section. One strip was incubated with a pooled serum sample that had not been pre-incubated with *A. dorsata* venom and was used as the reference strip. The scanned images of the blots were analyzed using ImageJ software (National Institutes of Health, NIH, 2004) and percentage inhibition was estimated.

### Measurement of IgE using Phadia ImmunoCAP test

IgE cross-reactivity to the crude venom of *V. vulgaris* and two recombinant components, rVes v1 (Phospholipase A_1_) and rVes v5 (Antigen 5) were evaluated using Phadia ImmunoCAP using the Phadia 100 instrument. Double-positivity was detected using *A. mellifera* crude ImmunoCAP. Four healthy control sera were used as controls. The test was performed according to the manufacturer’s instructions. The patients who had positive IgEto both *V. vulgaris* crude venom (or either of its two components) and *A. mellifera* crude venom were considered as double-positives. The samples which had double-positivity were pooled into three categories according to the IgE positivity to *A. mellifera* (bee) venom; Category 1—low IgE (n = 3; 0.35 − 3.4 kU_A_/l) positive, Category 2—moderate IgE (n = 16; 3.5 − 17.4 kU_A_/l) positive and category 3—high IgE (n = 4; 17.5 − 50 kU_A_/l) positive.

### Preparation of passive immune basophils from healthy donors

An aliquot of 100 µl of heparinized whole blood from a healthy donor was used in each test sample. Membrane bound IgE in donor basophils were removed using a minor modification of a previously described method [30]. Briefly, each blood sample was incubated for 15 min with 500 µl of Sodium lactate solution containing 14 mM Sodium lactate, 145 mM of NaCl and 6 mM KCl. The blood samples were centrifuged at 1200 *g* for 5 min at 4 °C. The supernatant was discarded carefully and the pellet was resuspended in 100 µl of 0.13 M PBS. The samples were incubated for 60 min at room temperature (25 °C) with 50 µl of serum of either *A. dorsata* venom allergic patients or patients having non-hymenopteran venom allergies (IgE control sera).

### Assessing basophil response to different venom concentrations

The test was performed according to the manufacturer’s instructions (BASOTEST™). Briefly, tubes containing 150 µl of processed donor basophils (100 µl of donor basophils and 50 µl of one patient’s serum—in one test) were initially incubated with stimulation buffer (provided with the kit) for 10 min at 37 °C. To test the most suitable concentration of *V. affinis* venom to be used in the passive BAT, the tubes were then incubated for 20 min at 37 °C with 1000 µl of three *V. affinis* venom concentrations (10 ng/ml, 100 ng/ml and 1 µg/ml). Degranulated basophils were stained using a 20 µl mixture of anti IgE-PE and anti CD63-FITC provided with the kit for 20 min on ice. The dose response was determined using the results.

### Gating strategy

Lymphocyte/basophil fraction was first selected by gating side-scatter vs forward scatter. The basophils were selected by gating the side scatter-low and IgE-high cell population. The samples were analyzed acquiring at least 200 basophils [25] using a FACSCalibur flow cytometer (BD Bioscience). The percentage activation of CD63 (basophil population with anti CD63-FITC intensity > 10^2^) was then calculated from the gated population for the different positive controls, negative controls and *V. affinis* venom.

### Passive basophil activation test using the patient serum

The passive BAT was carried out with serum samples from the 30 patients. Two positive controls were used: polyclonal anti-IgE (Sigma-Aldrich, Germany) at 3 dilutions, 1:10, 1: 100 and 1:1000 and fMLP provided with the kit (100 µl of 0.002 mM/ml) as per manufacturer’s instructions. Two groups of negative controls were used in the test; (i) donor basophils (without replacing IgE) were incubated in duplicate with 100 µl of the wash buffer and (ii) control sera from 7 patients who had non-hymenopteran venom allergy were added to donor basophils. Following incubation, the optimum concentration of *V. affinis* venom identified previously was added to each tube containing serum and incubated for 20 min at 37 °C. Activated basophils were stained for 20 min on ice using a 20 µl mixture of anti IgE-PE and anti CD63-FITC provided with the kit. Three categories of double IgE positives (by Phadia ImmunoCAP) were tested using the passive BAT.

### Determination of donor dependency

Two tests were performed using basophils from a second donor to determine the test performance of basophils from a different donor. Sera from two patients (patient# 5 and 10) were incubated with the IgE depleted basophils of the second donor. These were then incubated with *V. affinis* venom (1 µg/ml) and percentage CD63 activation was determined.

### Determination of the cutoff

The Cutoff in the passive BAT was set as a mean of negative controls + 3.3 SD [39]. Further, the stimulation index (SI) of each patient was calculated using the following formula; SI = percent of basophil activation of the test/mean percent of basophil activation of negative controls and any test with SI over 2 was considered as a positive.

### Statistical analysis

Statistical analysis was carried out using SPSS package (IBM, version 20). IgE positivity of crude venom of *V. affinis* and Ves v5 in ImmunoCAP was compared. Mann–Whitney U test was used in this comparison since the data was not normally distributed. Statistically significant level was P < 0.05 at 95% confidence level unless otherwise stated.

## Results

### Identification of *V. affinis* and venom extraction

**Fig. 2:**
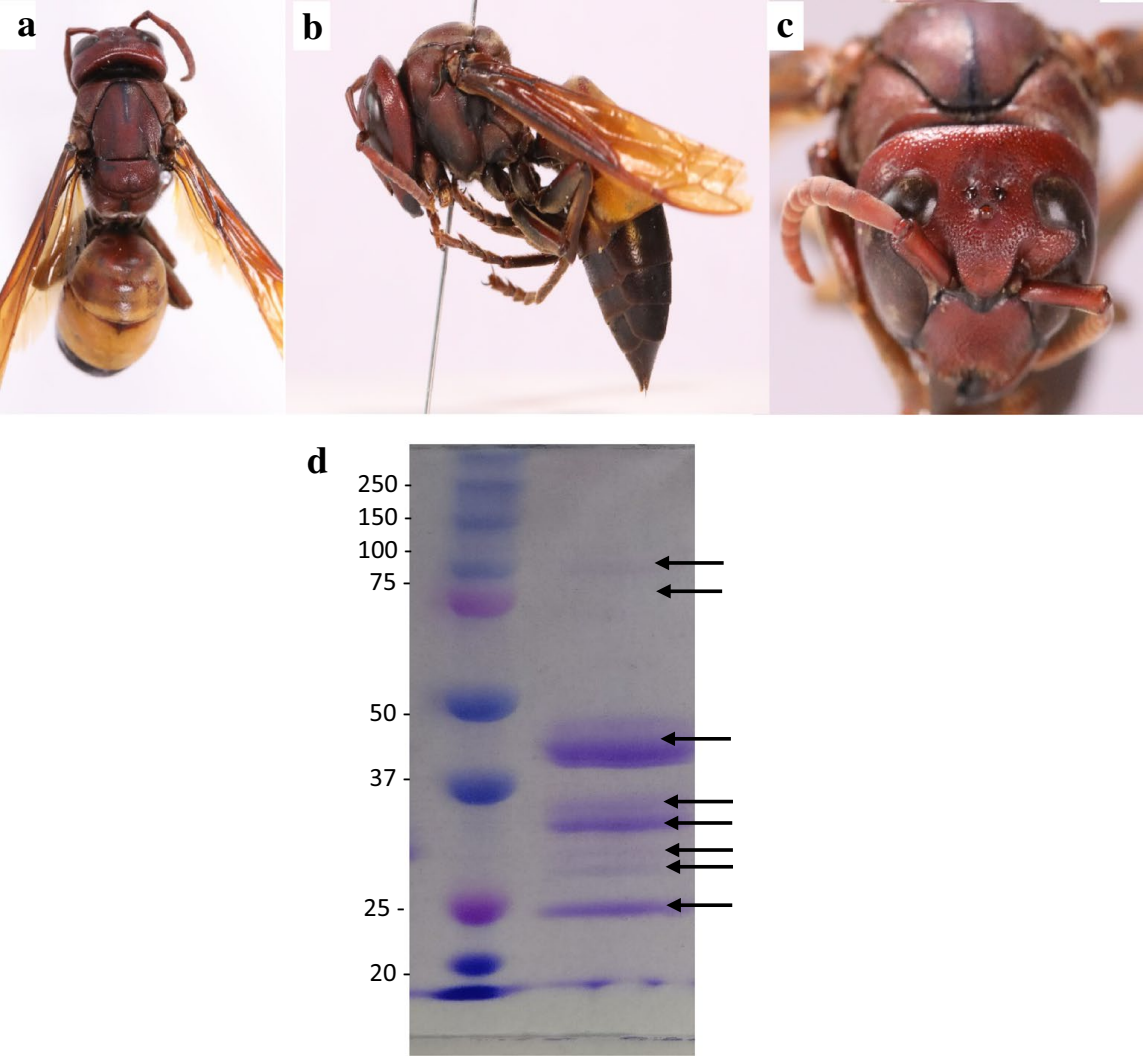
Morphology of *V. affinis* worker, **a** (i) ventral view, (ii) lateral view—the presence of orange or red metasomal segments I and II followed by posterior black segmentsand, (iii) red coloured face of *V. affinis* worker and **b** SDS-PAGE of *V. affinis* venom; Protein bands of 100, 80, 45, 38, 34, 28, 26, 24 kDa were detected. Lane 1—Maker and Lane 2—SDS-PAGE of *V. affinis* venom (30 µg)

*V. affinis* was identified by the characteristic dorsal, yellowish orange band on the first and second gastral tergites; an image of the specimen is shown in Fig. 2a.

### Immunochemical characterization allergens of *V. affinis*

**Fig. 3:**
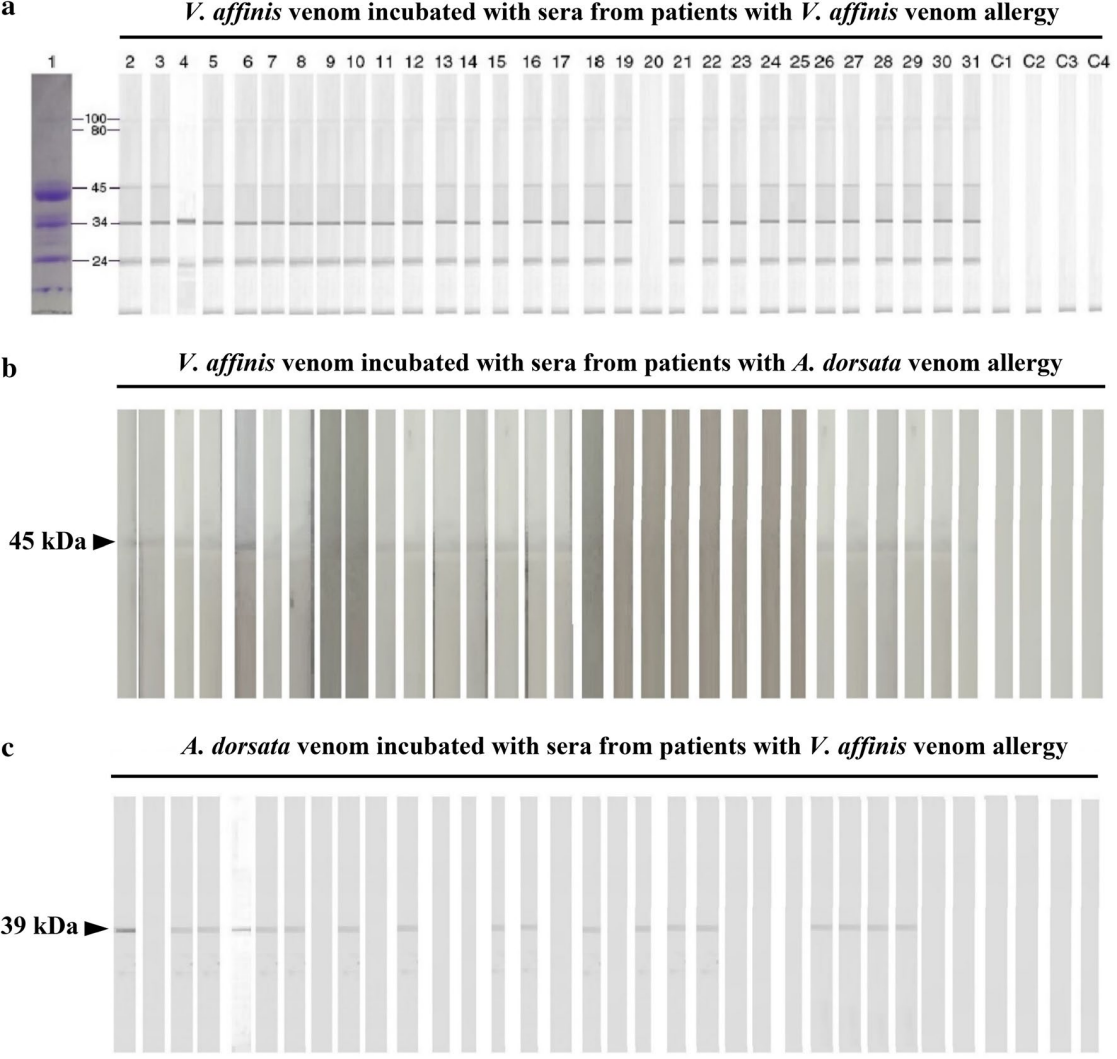
IgE reactivity in immunoblot. **a** *V. affinis* venom incubated with sera from the patients who were allergic to *V. affinis* venom, **b** *V. affinis* venom incubated with sera from the patients who were allergic to *A. dorsata* venom; 45 kDa allergen reacted with 16 of 30 sera and **c** *A. dorsata* venom incubated with sera from the patients who were allergic to *V. affinis* venom; 39 kDa allergen reacted with 18 of 30 sera: None of the healthy person’s sera reacted with either *V. affinis* or *A. dorsata* venom. Lane 1—SDS-PAGE of *V. affinis* venom, Lane 2 to 31—patients’ sera, Lane C1 to C4—healthy control sera

Eight proteins were visualized on SDS-PAGE, with approximate molecular weights of 100, 80, 45, 38, 34, 28, 26 and 24 kDa (Fig. 2b). Of these eight proteins, five (100, 80, 45, 34 and 24 kDa) showed reactivity with serum IgE in the immunoblots. Of the 30 patients, 29 (96%) had serum IgE reactivity with 34 and 24 kDa allergens of *V. affinis,* 28 (93%) reacted with the 45 kDa allergen, and 27 (90%) reacted with the 100 and 80 kDa allergens (Fig. 3a). None of the healthy control serum samples had IgE reactivity with *V. affinis* venom components.

### Identification of hyaluronidase of *V. affinis* venom

Of the 30 patients who had allergy to *V. affinis* venom, IgE reactivity to its 45 kDa band was detected in 93% of the sera (28/30) (Fig. 3a) whereas of 30 patients who had a history of allergy to *A. dorsata* venom, 53% of the sera (16/30) had IgE reactivity to the 45 kDa band (Fig. 3b). Conversely, 60% of the sera from *V affinis* allergy patients (18/30) showed IgE reactivity to a single band (the 39 kDa band) with an immunoblot of *A dorsata* venom, previously identified as hyaluronidase of *A. dorsata* (Fig. 3c).

**Fig. 4:**
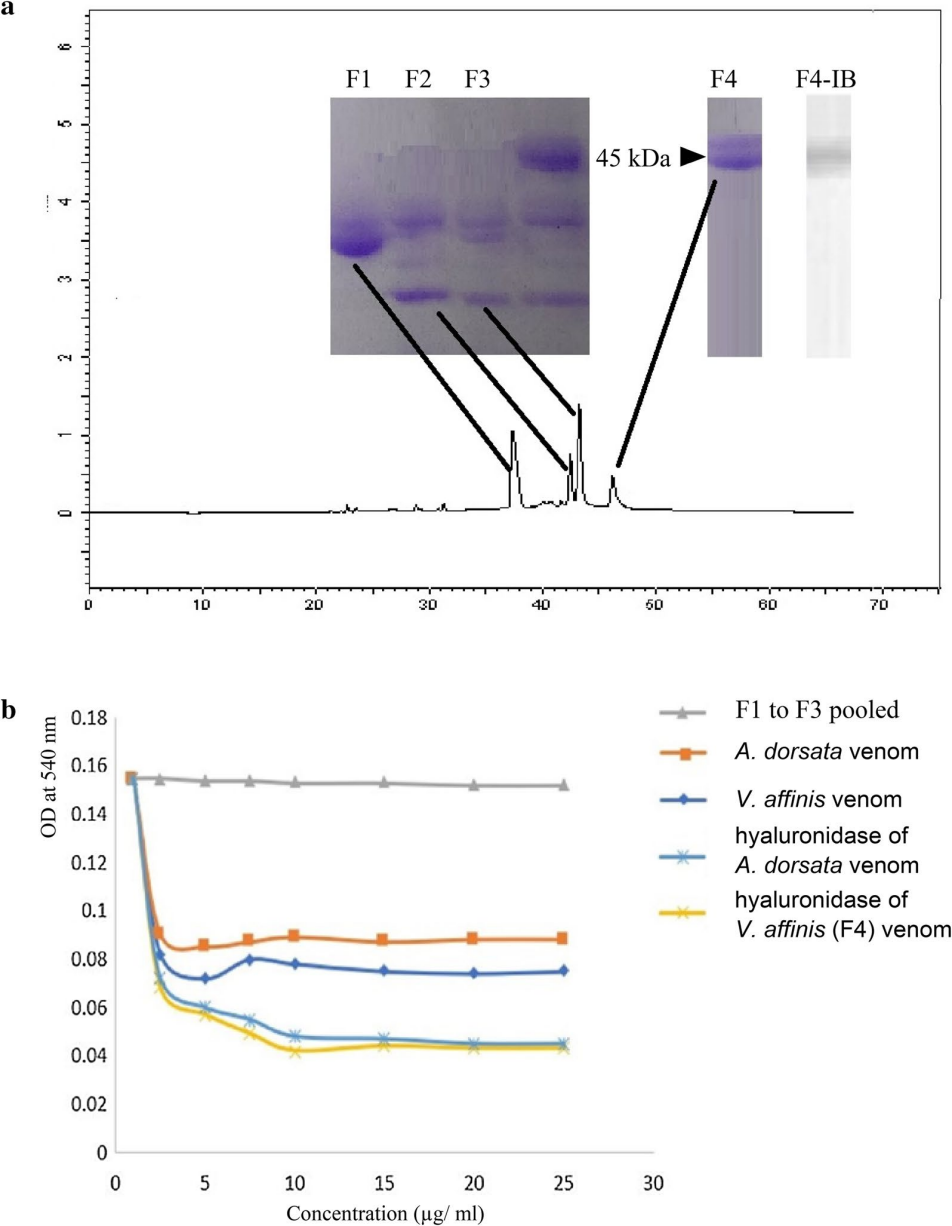
Identification and purification of hyaluronidase from *V. affinis* venom and comparison of its activity with hyaluronidase of *A. dorsata* venom: **a** venom profile obtained from high performance liquid chromatography (HPLC); F1 to F3—SDS-PAGE of the fractions eluted, CV—SDS-PAGE of crude *V. affinis* venom, F4—SDS-PAGE of hyaluronidase fraction of *V. affinis* venom and F4 IB—IgE reactivity to eluted F4 fraction in immunoblot and **b** comparison of hyaluronidase enzyme activity in *V. affinis* venom, *A. dorsata* venom and hyaluronidase fractions eluted from *V. affinis* and *A. dorsata* venom

Four major peaks were observed in the HPLC chromatogram of *V. affinis* venom (Fig. 4a) and these fractions were evaluated for hyaluronidase activity. Of the four major fractions (F1 to F4) eluted, only F4 which comprised the 45 kDa allergen on immunoblot, showed hyaluronidase activity confirming this allergen as hyaluronidase (Fig. 4b).

### Activity and allergenicity of hyaluronidase in *V. affinis* and *A. dorsata* venom

**Fig. 5:**
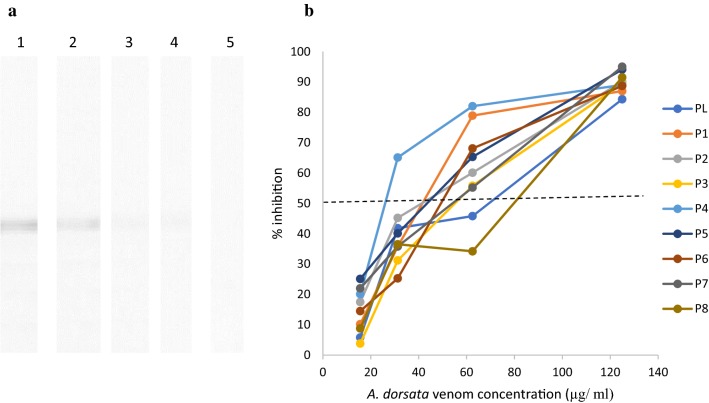
Immunoblot inhibition test; **a** inhibition with different *A. dorsata* venom concentrations (Lane 1—0, Lane 2—15.0.62, Lane 3—31.25, Lane 4—62.5 and Lane 5—125 µg/ml) with pooled sera **b** percentage inhibition of hyaluronidase *V. affinis* venom in 8 individual patients (P1 to 8) and pooled sera (PL); 50% inhibition is shown by the dotted line

Higher hyaluronidase activity (0.072 at 5 µg/ml) was seen with *V. affinis* crude venom compared to *A. dorsata* crude venom (0.085 at 5 µg/ml). However, both hyaluronidase fractions collected from *V. affinis* venom and *A. dorsata* venom have shown a similar hyaluronidase activity (0.060 at 5 µg/ml and stabilized in 0.045 at 17 µg/ml) (Fig. 4b). In immunoblot inhibition, pre-incubation of pooled serum with 125 µg/ml of *A. dorsata* venom showed an 87% inhibition *V. affinis* hyaluronidase (Fig. 5), confirming cross-reactivity between the 2 hyaluronidase fractions.

### Detection of IgE by Phadia ImmunoCAP

Of the 30 patients’ serum samples that were tested by Phadia ImmunoCAP, 13 (43%) had IgE to *V. vulgaris* venom, only one patient had IgE to Ves v1 whereas 22 (73%) had IgE to Ves v5. IgE positivity to Ves v5 was significantly different compared to that of crude venom (p = 0.02; Mann–Whitney U test) (Additional file 1: Figure S1). In addition, 23 of the 30 patients (76%) had IgE to *A. mellifera* (Western honeybee) crude venom by Phadia ImmunoCAP.

### Depletion of donor IgE from basophils and patient IgE attachment to treated-donor basophils

The removal of IgE attached to donor basophils following lactic acid treatment and attachment of patient IgE on the basophils after incubation with patient sera were confirmed by flow cytometry. The depletion of IgE attached to donor basophils following lactic acid treatment and the attachment of the patient’s IgE after incubation of treated basophils with patient sera is presented in Additional file 1: Figure S2.

### Selection of the suitable venom concentration

**Fig. 6:**
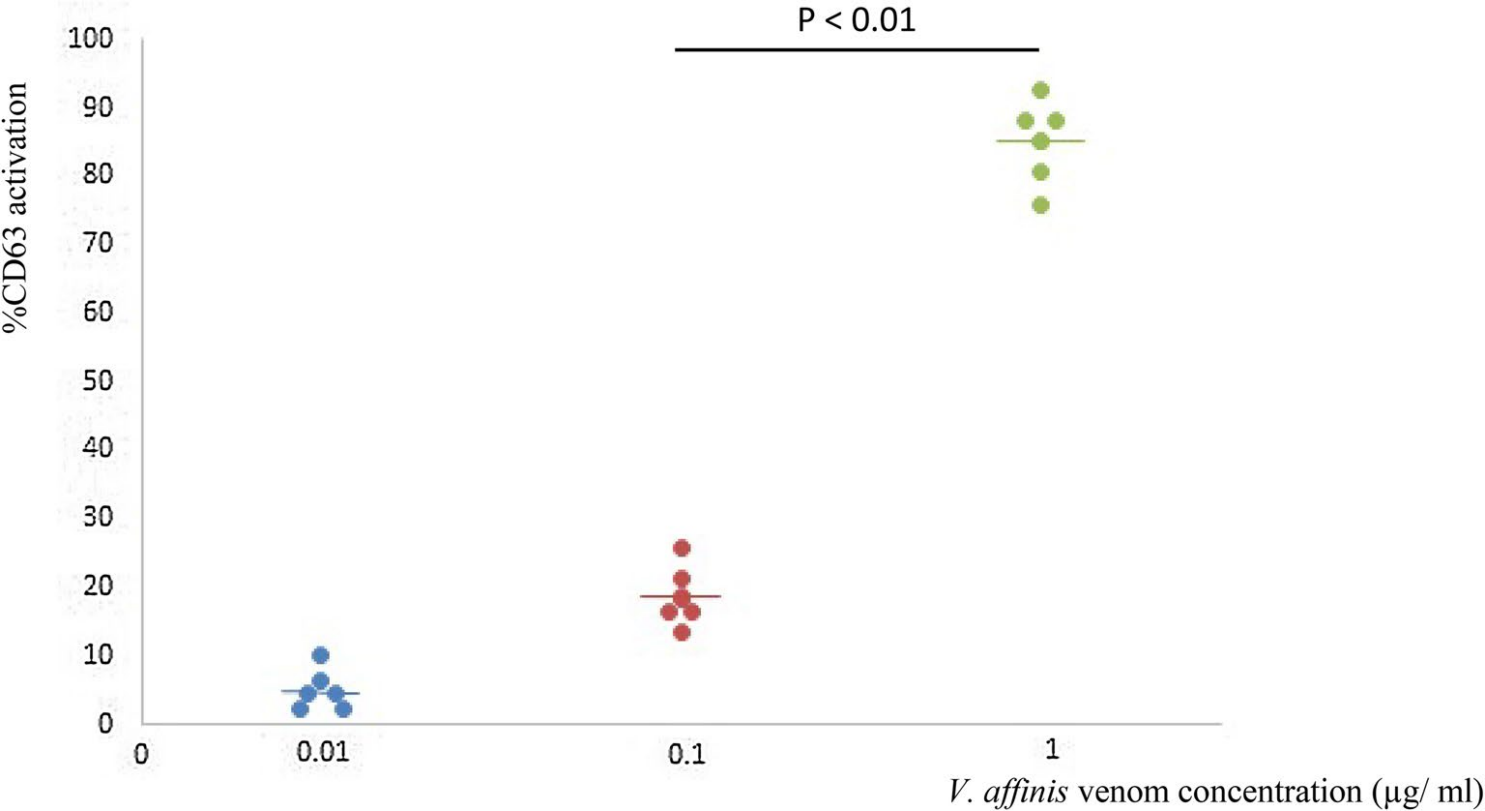
Assessing basophil response to different venom concentration to determine a suitable venom concentration to incubate the test samples (venom specific IgE levels to Ves v 5 of *V. vulgaris* in selected patients were 0.37, 0.52, 0.6, 0.62, 1.44 and 1.58 kU_A_/l)

Of the three-venom concentrations tested (10 ng/ml, 100 ng/ml, 1 µg/ml) using serafrom six patients, 1 µg/ml of *V. affinis* venom showed mean activation of 85% of basophils (Fig. 6 and data from previous studies [25, 40–42] were also taken into consideration when choosing the venom concentration) and this 1 µg/ml venom concentration was selected as the suitable venom concentration for further experiments.

### Passive immune basophil activation in response to *V. affinis* venom

**Fig. 7:**
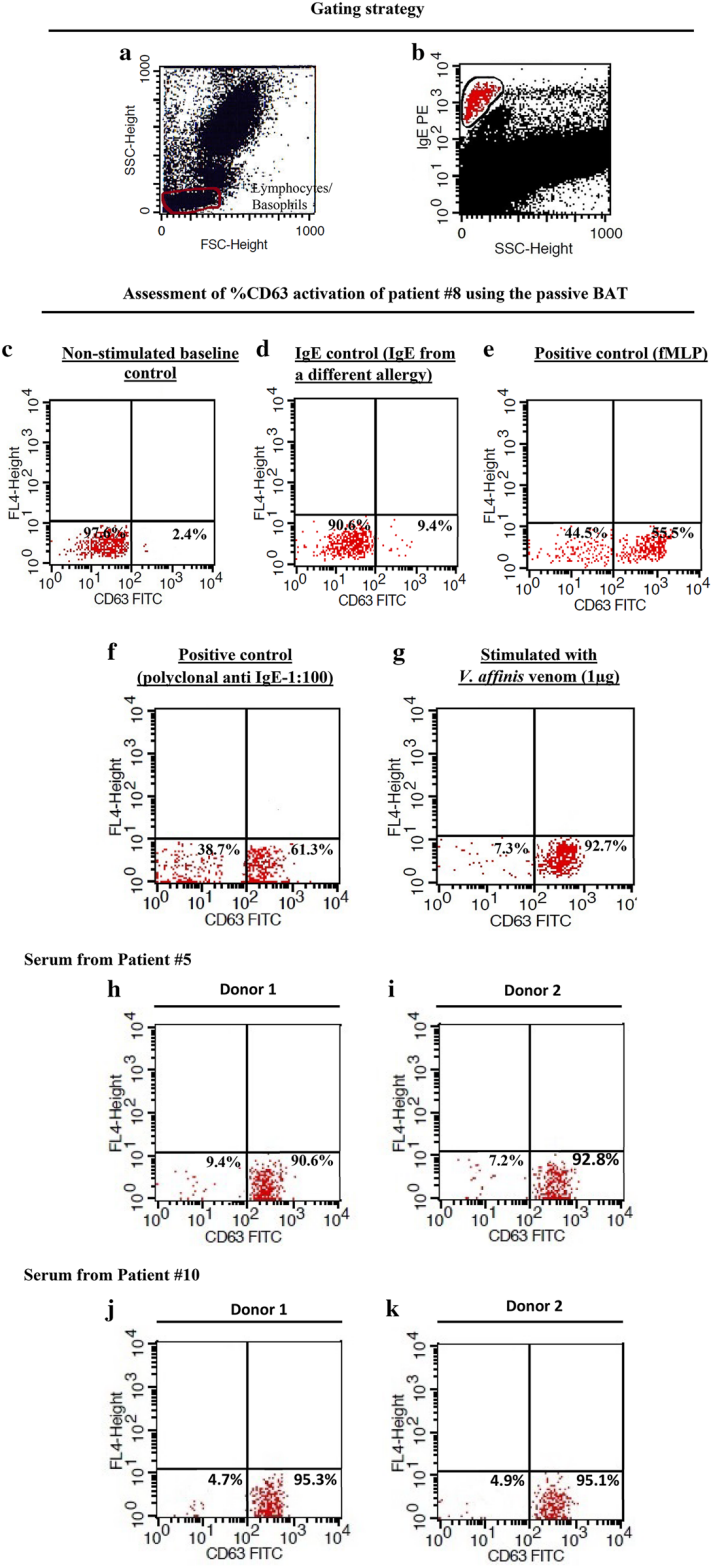
Gating strategy, evaluation of a patient serumand donor dependency of the passive BAT; **a** Basophils were gated in the lymphocyte region of the SSC/FSC pattern **b** selected high IgE^pos^ population, **c** non-stimulated baseline control, **d** IgE control—stimulated with a different IgE source, **e** positive control (fMLP), **f** positive control (polyclonal anti IgE), **g** evaluation of %CD63 activation by *V. affinis* venom − 1 µg, **h**–**k** basophils from two donors (donor 1 and 2) have been given a similar activation after incubation with sera from patient #5 and sera from patient #10

Initial gating strategies to select the IgE high basophil population is shown in Fig. 7a, b. The negative control which was incubated only with stimulation buffer resulted in < 5% of basophil activation (Fig. 7c). The negative control with basophils incubated with sera from non-hymenoptera allergic patients had < 10% basophil activation in response to *V. affinis* venom (Fig. 7d). The positive controls incubated with fLMP had a basophil activation of 55.5% (Fig. 7e). Polyclonal anti-IgE at a dilution of 1: 100 gave a basophil activation of 61.2% (Fig. 7f). Percentage CD63 activation of the basophils was 92.7% with patient #8 and is shown in Fig. 7g.

### Determination of donor dependency of the test

Sera from patients #5 and #10 showed similar activation with basophils from both donors (Fig. 7h–k).

### Cutoff selection and interpretation of the test results

The cutoff was selected as 13% activation of basophils. Of the 30 patients with anaphylaxis to *V. affinis* venom, all 30 (100%) were positive to *V affinis* venom in the passive BAT; 100% had over 45% activation, 93% (28/30) had over 60% activation and 73% (22/30) had over 80% activation and all had SI over 2. The donor basophils were not activated by the sera of double-positive patients (n = 23, pooled into 3 groups) after incubation with the venom of *A. dorsata*.

## Discussion

Five IgE reactive allergens (with molecular weights of 100, 80, 45, 34 and 24 kDa) were identified by immunoblots from *V. affinis* venom, using the sera of 30 *V. affinis* allergic adults. Of these allergens, the 45 kDa band was identified as hyaluronidase. The 34 kDa band was previously reported as phospholipase A_1_ [2, 43] and a 24 kDa band was identified as antigen 5 in the venom of *Vespula vulgaris* (Western wasp), *Vespa crabro* (Western hornet) and *P. paulista* (wasp prevalent in the South American region) [28, 43–45] and a 100 kDa band was identified as dipeptidylpeptidase IV in the venom of *V. vulgaris* [28, 45] thus, the 24 kDa and the 100 kDa allergens identified in our study may be analogues to antigen 5 and dipeptidylpeptidase IV respectively, of *V. affinis*. At present, the identity of the 80 kDa allergen is uncertain. The high IgE reactivity for the five allergens of *V. affinis* points to the possibility of using these allergens in component resolved diagnosis (CRD) of *V*. *affinis* venom allergy.

The allergen profile of *V. affinis* observed by immunoblots in this study is similar to the allergen profile of *V. vulgaris* a Western wasp species of family Vespidae. However, the sensitivity of crude *V. vulgaris* venom and Ves v1 by Phadia Immunocap was very low in our patients. Thus, the use of crude venom and Ves v1 of *V. vulgaris* in the diagnosis of *V. affinis* venom allergy may be limited. The higher IgE reactivity to Ves v5 (73%) and low reactivity to crude venom of *V. vulgaris* may indicate that the amount of Ves v5 in crude venom of *V. vulgaris* is minimal and in line with previous studies which have shown low amounts of Ves v5 in *V. vulgaris* venom [45, 46].

Hyaluronidase was one of the major allergenic components of *V. affinis* venom in the present study. We have previously identified hyaluronidase as a major allergen in *A. dorsata* (Giant Asian Honeybee) venom allergy [30]. In our study, IgE cross-reactivity to hyaluronidase was similar in these two species (60% in *V affinis* venom and 53% in *A. dorsata* venom respectively). Hyaluronidase is highly conserved in many species [28, 47] and cross-reactivity of hyaluronidase between honeybees and wasp species prevalent in Western countries had been demonstrated previously [48, 49].

We found the passive basophil activation test (BAT) to be a good method to diagnose the venom allergy. In the present study, we used the passive BAT to diagnose of *V. affinis* venom allergy. To the best of our knowledge, this is the first report which shows the utility of the passive BAT in the diagnosis of hymenoptera venom allergy. The passive BAT has been previously used to detect transfusion reactions in two patients [27] Wilson et al. [50] postulated the use of passive BAT for the diagnose of α-Gal allergy for a better understanding of the ability to activate donor basophils by patients’ sera, but the concept was not tested.

In the present study, we determined an optimal concentration of the venom initially using sera from six patients. This concentration (1 µg/ml) was then used to test the rest of the samples. While some studies use only one concentration of the allergen after obtaining the most suitable concentration [25, 40–42] as 1 µg/ml, others use different concentrations with each patient. The rationale is that the sensitivity to the allergen may vary from patient to patient, and therefore the basophils from some patients may not get activated with the chosen concentration. The sensitivity may vary due to the serum IgE concentration, quality of the antibody, or due to the sensitivity of the basophils of each patient. As we are using the basophils of a single non-atopic donor, the sensitivity of the basophils will not vary between patients, and we believe that it is not necessary to use multiple concentrations of venom. In addition, using this concentration, 100% had a basophil activation of > 45%, which was > 4 times that of the negative control.

Studies have found that up to 20% of people have basophils which are not activated by allergens [51, 52] despite the presence of allergen-specific IgE. However, we used basophils from a single donor to test all our patients. It is noteworthy that basophils from a non-responder could impede the test. In these situations, it is advisable to select the right donor through an initial evaluation of the basophils. Further, a reduction of 80% of basophils immediately after anaphylaxis [53] may impinge upon the performance of conventional BAT whereas it would not affect the performance of passive BAT which was used in the present study.

Results of the present study showed that all patients who developed anaphylaxis to *V. affinis* had a positive BAT. Reported sensitivity and specificity, in conventional BAT, using *Vespula vulgaris* (closely related species to *Vespa affinis*) were within 60–80% [25, 54] in patients who had systemic allergic reactions (patients with the systemic reactions of Muller grade II–IV). Also, sensitivity and specificity of 85–100% were reported [40–42] in the patients who had anaphylaxis (patients with at least one systemic reaction of Muller grade III–IV) to hymenopteran venom and the results are comparable with the results of the present study.

Both immunoblot and passive BAT had comparably high positivity rates of 96% and 100% respectively. The observed cross-reactivity for hyaluronidase in the immunoblots is likely due to cross-reactive carbohydrate determinants (CCD) whereas the passive BAT shows no positivity with bee venom.

In vitro testing has indicated double-positivity with both bee and wasp venom sensitivity in patients allergic to one species on many occasions [28, 29]. This may be due to allergy to both species, true cross-reactivity or due to clinically innocuous CCD [28, 29]. By ImmunoCAP, the positivity rate for bee venom in patients allergic to *V. affinis* venom (double-positivity) was 76%. In contrast, passive BAT shows no positivity with bee venom. Passive BAT has a significant advantage in the identification of the culprit insect compared to the Phadia ImmunoCAP and may avoid misidentification. However, passive BAT revealed an absence of cross-reactivity, in keeping with other studies which indicate that the conventional BAT is useful in differentiating true-positivity from false double-positivity particularly when used CD63 as activation marker [40–42, 55, 56]. However, one study [57] has found 50–67% of patients with double-positivity (through cross-reactive carbohydrate determinants) in conventional BAT, in contrast to the present study. The plausible reason may be the basophil activation marker (CD203c) that was used, could be upregulated and expressed in response to many non-degranulation stimuli (piecemeal activation) whereas in our study we have used CD63 as the activation maker which is upregulated only during anaphylaxis [58, 59].

Passive BAT which was used in the present study has advantages over conventional BAT. Firstly, blood samples need to be processed within 4 h in conventional BAT for best performance [25]. However, in areas where it is difficult to get samples to specialized laboratories (e.g.; rural Sri Lanka), it is not possible to perform the test within the required time frame. On the other hand, collection and storage of serum samples are feasible, to be sent to a central laboratory for processing and flow cytometry.

Around 4–6% of patients who are allergic to wasps/bees have negative results with skin prick test (SPT)/intradermal (ID) test or Phadia ImmunoCAP test [60] and such patients may need a challenge test for further evaluation. The patients who gave negative results with Phadia ImmunoCAP, (including one patient who had a negative immunoblot result as well), was identified by the passive BAT as being sensitive to hornet venom. These patients may not need challenge tests to identify venom allergy. Hence, passive BAT may be used in lieu of challenge tests, which are potentially dangerous. Rarely, SPT/ID tests may give rise to systemic reactions, especially in patients who had developed severe reactions to insect stings. Passive BAT may be a suitable alternative in such instances.

In conclusion, we found five allergens in *V. affinis* venom and these may be suitable candidates for future CRD of *V. affinis* venom allergy. Cross-reactivity to hyaluronidase of *V. affinis* and *A. dorsata* was shown in our study; however, this was not due to true cross-reactivity (due to CCD). Hence, hyaluronidase would not be a suitable candidate for differentiating *V. affinis* from *A. dorsata* venom allergy. The passive BAT has shown its importance as a promising diagnostic tool with high accuracy. It would be particularly useful in cases with doubtful double-positive results of other diagnostic tests or unclear clinical history.

## Supplementary information

**Additional file 1: Figure S1.** Comparison of IgE reactivity; Specific IgE to crude venom of *V. vulgaris* and Ves v 5 (*P *=* 0.02* in Mann–Whitney U test) Horizontal bars indicate the mean of specific IgE quantity to either *V. vulgaris* venom or Ves v 5 and the dotted line represent the Phadia ImmunoCAP cut off level 0.1 kU_A_/l. **Figure S2.** Generation of passive immune donor basophils; (a) membrane bound IgE on donor basophils (b) removal of donor IgE by lactic acid treatment and (c) reattachment of patient IgE; top—scatter diagram and bottom—intensity diagram.

## Data Availability

The datasets analyzed during the current study are available from the corresponding author on a reasonable request.

## Abbreviations

CCD: cross-reactive carbohydrate determinants
SDS-PAGE: sodium dodecyl sulphate gel electrophoresis
HPLC: high-performance liquid chromatography
PBS: phosphate buffered saline
BSA: bovine serum albumin
BAT: basophil activation test
SI: stimulation index
SPT: skin prick test
ID: intradermal test
CD: cluster of differentiation

## References

[1] Jeyakanth T, Mayurathan P, Sivansuthan S (2015). Lower limb ischemia and multiple organ dysfunction syndrome following wasp sting. Anuradhapura Med J.

[2] Sookrung N, Wong-din-dam S, Tungtrongchitr A, Reamtong O, Indrawattana N, Sakolvaree Y, Visitsunthorn N, Manuyakorn W, Chaicumpa W (2014). Proteome and allergenome of Asian wasp, *Vespa affinis*, venom and IgE reactivity of the venom components. J Proteome Res.

[3] *Vespa affinis* (Linnaeus, 1764) in GBIF Secretariat. GBIF Backbone Taxonomy. Checklist dataset; 2017. 10.15468/39omei. Accessed via GBIF.org on 02 Dec 2018.

[4] *Vespula vulgaris* (Linnaeus, 1758) in GBIF Secretariat. GBIF Backbone Taxonomy. Checklist dataset; 2017. 10.15468/39omei. Accessed via GBIF.org on 02 Dec 2018.

[5] Witharana EWRA, Wijesinghe SK, Pradeepa KS, Karunaratne WA, Jayasinghe S (2015). Bee and wasp stings in Deniyaya; a series of 322 cases. Ceylon Med J.

[6] Kularatne SAM, Shahmy S, Rathnayake SS, Dawson AH (2018). Clinico-epidemiology of arthropod stings and bites in primary hospitals of North Western province of Sri Lanka. Clin Toxicol.

[7] George P, Pawar B, Calton N, Mathew P (2008). Wasp sting: an unusual fatal outcome. Saudi J Kidney Dis Transpl.

[8] Xuan BH, Mai HL, Thi TX, Thi MT, Nguyen HN, Rabenou RA (2010). Swarming hornet attacks: shock and acute kidney injury–a large case series from Vietnam. Nephrol Dial Transplant.

[9] Kar S, Dongre A, Krishnan A, Godse S, Singh N (2013). Epidemiological study of insect bite reactions from central India. Indian J Dermatol.

[10] Lama T, Karmacharya B, Chandler C, Patterson V (2011). Telephone management of severe wasp stings in rural Nepal: a case report. J Telemed Telecare.

[11] Kularatne K, Kannangare T, Jayasena A, Jayasekera A, Waduge R, Weerakoon K, Kularatne SA (2014). Fatal acute pulmonary oedema and acute renal failure following multiple wasp/hornet (*Vespa affinis*) stings in Sri Lanka: two case reports. J Med Case Rep.

[12] Ralapanawa DM, Kularatne SA (2014). A case of Kounis syndrome after a hornet sting and literature review. BMC Res Notes.

[13] Wijerathne BT, Rathnayake GK, Agampodi SB (2014). Hornet stings presenting to a primary care hospital in Anuradhapura District, Sri Lanka. Wild Environ Med.

[14] Lee HL, Krishnasamy M, Jeffery J (2005). A fatal case of anaphylactic shock caused by the lesser banded hornet, *Vespa affinis* indosinensis in peninsular Malaysia. Trop Biomed.

[15] Thong BY, Cheng YK, Leong KP, Tang CY, Chng HH (2005). Anaphylaxis in adults referred to a clinical immunology/allergy centre in Singapore. Singapore Med J.

[16] Paudel B, Paudel K (2009). A study of wasp bites in a tertiary hospital of western Nepal. Nepal Med Coll J.

[17] Rungsa P, Incamnoi P, Sukprasert S, Uawonggul N, Klaynongsruang S, Daduang J, Patramanon R, Roytrakul S, Daduang S (2016). Comparative proteomic analysis of two wasps venom, *Vespa tropica* and *Vespa affinis*. Toxicon.

[18] Nandi M, Sarkar S (2012). Acute kidney injury following multiple wasp stings. Pediatr Nephrol.

[19] Pramanik S, Banerjee S (2007). Wasp stings with multisystem dysfunction. Indian Pediatr.

[20] Dhanapriya J, Dineshkumar T, Sakthirajan R, Shankar P, Gopalakrishnan N, Balasubramaniyan T (2016). Wasp sting-induced acute kidney injury. Clin Kidney J.

[21] Sigdel MR, Raut KB (2013). Wasp bite in a referral hospital in Nepal. J Nepal Health Res Counc.

[22] Vishwanath P, Adhikari DA, Poudel R, Poudel K, Pillay V, Menezes R (2010). Myocarditis and mobitz type I heart block following wasp sting. Internet J Cardiol.

[23] Ullah P, Chowdhury A, Isha IT, Mahmood S, Chowdhury RF, Zeesan-ul-Abir M, Manna AA, Patwary MI (2016). Wasp stings (*Vespa affinis*) induced acute kidney injury following rhabdomyolysis in a 25-year-old woman. J Emerg Pract Trauma.

[24] Chowdhury FR, Bari MS, Shafi AM, Ruhan AM, Hossain ME, Chowdhury S, Hafiz MA (2014). Acute kidney injury following Rhabdomyolysis due to multiple wasp stings (*Vespa affinis*). Asia Pac J Med Toxicol.

[25] Sturm GJ, Böhm E, Trummer M, Weiglhofer I, Heinemann A, Aberer W (2004). The CD63 basophil activation test in Hymenoptera venom allergy: a prospective study. Allergy.

[26] Pruzansky JJ, Grammer LC, Patterson R, Roberts M (1983). Dissociation of IgE from receptors on human basophils. I. Enhanced passive sensitization for histamine release. J Immunol.

[27] Yasui K, Matsuyama N, Okamura-Shiki I, Ikeda T, Ishii K, Furuta RA, Hirayama F (2017). Clinical utility of a passive immune basophil activation test for the analysis of allergic transfusion reactions. Transfusion.

[28] Hemmer W, Focke M, Kolarich D, Wilson IB, Altmann F, Wöhrl S, Götz M, Jarisch R (2001). Antibody binding to venom carbohydrates is a frequent cause for double positivity to honeybee and yellow jacket venom in patients with stinging-insect allergy. J Allergy Clin Immunol.

[29] Ebo DG, Hagendorens MM, Bridts CH, De Clerck LS, Stevens WJ (2004). Sensitization to cross-reactive carbohydrate determinants and the ubiquitous protein profilin: mimickers of allergy. Clin Exp Allergy.

[30] Gunasekara P, Handunnetti SM, Premawansa S, Witharana EWRA, Dasanayake WMKD, Ratnayake IP, Seneviratne SL, Dias RKS, Premakumara GAS, de Silva R (2017). IgE cross-reactivity of phospholipase A_2_ and hyaluronidase of *Apis dorsata* (Giant Asian Honeybee) and *Apis mellifera* (Western Honeybee) venom: Possible use of *A. mellifera* venom for diagnosis of patients allergic to *A. dorsata* venom. Toxicon.

[31] Bilo BM, Rueff F, Mosbech H, Bonifazi F, Oude-Elberink JN (2005). EAACI interest group on insect venom hypersensitivity. Diagnosis of Hymenoptera venom allergy. Allergy.

[32] Perez-Riverol A, Miehe M, Jabs F, Seismman H, Fernandes LG, de Lima Zollner R, Jakob T, Braga MR, Spillner E (2018). Venoms of Neotropical wasps lack cross-reactive carbohydrate determinants enabling reliable protein-based specific IgE determination. J Allergy Clin Immunol.

[33] Caruso B, Bonadonna P, Severino MG, Manfredi M, Dama A, Schiappoli M, Rizzotti P, Senna G, Passalacqua G (2007). Evaluation of the IgE cross‐reactions among vespid venoms. A possible approach for the choice of immunotherapy. Allergy.

[34] Müller UR (2002). Recombinant Hymenoptera venom allergens. Allergy.

[35] Macchia D, Cortellini G, Mauro M, Meucci E, Quercia O, Manfredi M, Massolo A, Valentini M, Severino M, Passalacqua G (2018). *Vespa crabro* immunotherapy versus Vespula-venom immunotherapy in *Vespa crabro* allergy: a comparison study in field re-stings. World Allergy Organ J.

[36] Lu G, Gonzalez M, Qian N, Soldatova L (1996). Yellowjacket venom allergens, hyaluronidase and phospholipase: sequence similarity and antigenic cross-reactivity with their hornet and wasp homologs and possible implications for clinical allergy. J Allergy Clin Immunol.

[37] Kimsey LS, Carpenter JM (2012). The Vespinae of North America. (Vespidae; Hymenoptera). J Hymenop Res.

[38] Bradford MM (1976). A rapid and sensitive method for the quantitation of microgram quantities of protein utilizing the principle of protein-dye binding. Anal Biochem.

[39] Hamilton RG, Matsson PNJ, Adkinson FJ, Chan S, Hovanec-Burns D, Kleine-Tebbe J, et al. Analytical performance characteristic s, quality assurance, and clinical utility of immunological assays for human immunoglobulin e antibodies of defined allergen specificities. Wayne: Clinical and Laboratory Standards Institute; 2016 October. Report No.: CLSE Report I/LA20 Contract No.: 1.

[40] Eberlein-König B, Varga R, Mempel M, Darsow U, Behrendt H, Ring J (2006). Comparison of basophil activation tests using CD63 or CD203c expression in patients with insect venom allergy. Allergy.

[41] Korošec P, Šilar M, Eržen R, Čelesnik N, Bajrović N, Zidarn M, Košnik M (2013). Clinical routine utility of basophil activation testing for diagnosis of hymenoptera-allergic patients with emphasis on individuals with negative venom-specific IgE antibodies. Int Arch Allergy Immuno.

[42] Erdmann SM, Sachs B, Kwiecien R, Moll-Slodowy S, Sauer I, Merk HF (2004). The basophil activation test in wasp venom allergy: sensitivity, specificity and monitoring specific immunotherapy. Allergy.

[43] Sukprasert S, Rungsa P, Uawonggul N, Incamnoi P, Thammasirirak S, Daduang J, Daduang S (2013). Purification and structural characterisation of phospholipase A_1_ (Vespapase, Ves a 1) from Thai banded tiger wasp (*Vespa affinis*) venom. Toxicon.

[44] Hoffman DR (1993). Allergens in Hymenoptera venom XXV: the amino acid sequences of antigen 5 molecules and the structural basis of antigenic cross-reactivity. J Allergy Clin Immunol.

[45] Huss-Marp J, Raulf M, Jakob T (2015). Spiking with recombinant allergens to improve allergen extracts: benefits and limitations for the use in routine diagnostics. Allergo J Int.

[46] Vos B, Köhler J, Müller S, Stretz E, Ruëff F, Jakob T (2013). Spiking venom with rVes v 5 improves sensitivity of IgE detection in patients with allergy to Vespula venom. J Allergy Clin Immunol.

[47] An S, Chen L, Wei JF, Yang X, Ma D, Xu X, Xu X, He S, Lu J, Lai R (2012). Purification and characterization of two new allergens from the venom of *Vespa magnifica*. PLoS ONE.

[48] Blank S, Bilò MB, Ollert M (2018). Component-resolved diagnostics to direct in venom immunotherapy: important steps towards precision medicine. Clin Exp Allergy.

[49] Hemmer W (2014). Cross reactions between Hymenoptera venoms from different families, genera and species. Der Hautarzt; Zeitschrift fur Dermatologie, Venerologie, und verwandte Gebiete.

[50] Wilson JM, Platts-Mills TA (2019). IgE to galactose-α-1, 3-galactose and the α-Gal syndrome: insights from basophil activation testing. J Allergy Clin Immunol.

[51] Commins SP, James HR, Stevens W, Pochan SL, Land MH, King C, Mozzicato S, Platts-Mills TA (2014). Delayed clinical and ex vivo response to mammalian meat in patients with IgE to galactose-alpha-1, 3-galactose. J Allergy Clin Immunol.

[52] De Weck AL, Sanz ML, Gamboa PM, Ebo DG (2008). Diagnostic tests based on human basophils: more potentials and perspectives than pitfalls, 2: technical issues. J Investig Allergol Clin Immunol.

[53] Korosec P, Turner PJ, Silar M, Kopac P, Kosnik M, Gibbs BF, Shamji MH, Custovic A, Rijavec M (2017). Basophils, high-affinity IgE receptors, and CCL2 in human anaphylaxis. J Allergy Clin Immunol.

[54] Sainte-Laudy J, Sabbah A, Drouet M, Lauret MG, Loiry M (2000). Diagnosis of venom allergy by flow cytometry. Correlation with clinical history, skin tests, specific IgE, histamine and leukotriene C4 release. Clin Exp Allergy.

[55] Eberlein B, Krischan L, Darsow U, Ollert M, Ring J (2012). Double positivity to bee and wasp venom: improved diagnostic procedure by recombinant allergen–based IgE testing and basophil activation test including data about cross-reactive carbohydrate determinants. J Allergy Clin Immunol.

[56] Kosnik M, Korosec P (2009). Importance of basophil activation testing in insect venom allergy. Allergy Asthma Clin Immunol.

[57] Mertens M, Amler S, Moerschbacher BM, Brehler R (2010). Cross-reactive carbohydrate determinants strongly affect the results of the basophil activation test in hymenoptera-venom allergy. Clin Exp Allergy.

[58] MacGlashan D (2010). Expression of CD203c and CD63 in human basophils: relationship to differential regulation of piecemeal and anaphylactic degranulation processes. Clin Exp Allergy.

[59] Dahlén B, Nopp A, Johansson SG, Eduards M, Skedinger M, Adedoyin J (2011). Basophil allergen threshold sensitivity, CD-sens, is a measure of allergen sensitivity in asthma. Clin Exp Allergy.

[60] Korosec P, Erzen R, Silar M, Bajrovic N, Kopac P, Kosnik M (2009). Basophil responsiveness in patients with insect sting allergies and negative venom-specific immunoglobulin E and skin prick test results. Clin Exp Allergy.

