# Supplementary material for: Diagnosis of Vespa affinis venom allergy: use of immunochemical methods and a passive basophil activation test
Source: Allergy Asthma Clin Immunol. 2019 Dec 4;15:80. doi: 10.1186/s13223-019-0394-6 (PMC6894289; doi:10.1186/s13223-019-0394-6)
Supplement: Supplementary file 1 — Additional file 1: Figure S1. Comparison of IgE reactivity; Specific IgE to crude venom of V. vulgaris and Ves v 5 (P = 0.02 in Mann–Whitney U test) Horizontal bars indicate the mean of specific IgE quantity to either V. vulgaris venom or Ves v 5 and the dotted line represent the Phadia ImmunoCAP cut off level 0.1 kUA/l. Figure S2. Generation of passive immune donor basophils; (a) membrane bound IgE on donor basophils (b) removal of donor IgE by lactic acid treatment and (c) reattachment of patient IgE; top—scatter diagram and bottom—intensity diagram. [file 13223_2019_394_MOESM1_ESM.docx]

Figure S1


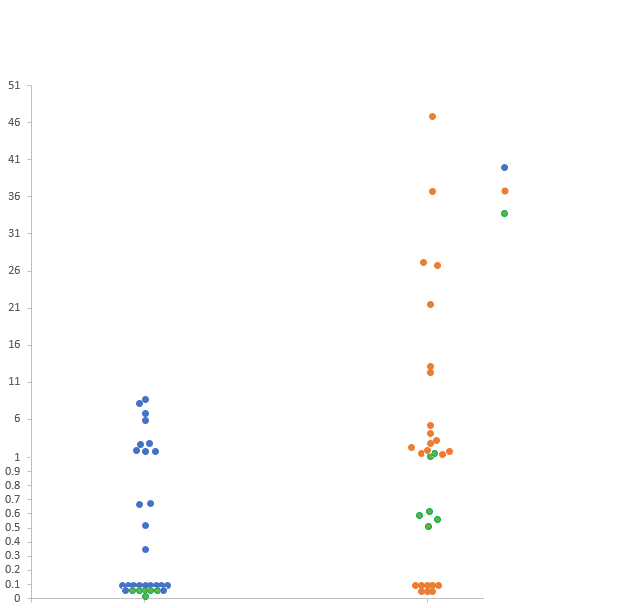


*Vespula vulgaris* crude venom

Ves v 5

Patients selected in pre-BAT

IgE quantity (KU_A_/L)

Figure S2


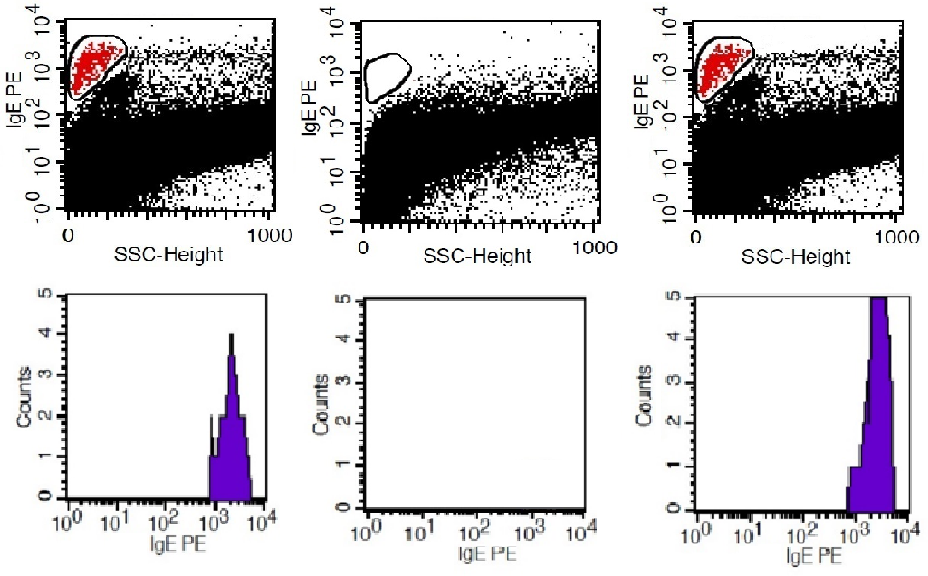


c

b

a
